# Supplementary material for: Multi‐dataset identification of innovative feature genes and molecular mechanisms in keratoconus
Source: J Cell Mol Med. 2024 Sep 19;28(18):e70079. doi: 10.1111/jcmm.70079 (PMC11412914; doi:10.1111/jcmm.70079)
Supplement: Supplementary file 2 — Material S2. [file JCMM-28-e70079-s002.zip › 7.GSEA analysis/4.MYRF/KEGG_ADIPOCYTOKINE_SIGNALING_PATHWAY.html]

Details for gene set KEGG\_ADIPOCYTOKINE\_SIGNALING\_PATHWAY[GSEA]

|  || Dataset | MYRF.group.cls#h\_versus\_l |
| Phenotype | group.cls#h\_versus\_l |
| Upregulated in class | l |
| GeneSet | KEGG\_ADIPOCYTOKINE\_SIGNALING\_PATHWAY |
| Enrichment Score (ES) | -0.4223226 |
| Normalized Enrichment Score (NES) | -1.5224049 |
| Nominal p-value | 0.051896207 |
| FDR q-value | 0.20876868 |
| FWER p-Value | 0.731 |
Table: GSEA Results Summary

  

Fig 1: Enrichment plot: KEGG\_ADIPOCYTOKINE\_SIGNALING\_PATHWAY      
 Profile of the Running ES Score & Positions of GeneSet Members on the Rank Ordered List

  

| SYMBOL | TITLE | RANK IN GENE LIST | RANK METRIC SCORE | RUNNING ES | CORE ENRICHMENT || 1 | LEP | na | 316 | 0.646 | 0.0480 | No |
| 2 | ADIPOQ | na | 2112 | 0.347 | 0.0269 | No |
| 3 | ACSL6 | na | 2596 | 0.318 | 0.0410 | No |
| 4 | IRS4 | na | 7035 | 0.163 | -0.0726 | No |
| 5 | CPT1A | na | 8327 | 0.131 | -0.0983 | No |
| 6 | MAPK9 | na | 8409 | 0.129 | -0.0892 | No |
| 7 | MTOR | na | 8431 | 0.129 | -0.0785 | No |
| 8 | CAMKK1 | na | 8636 | 0.124 | -0.0734 | No |
| 9 | PRKAG2 | na | 9896 | 0.098 | -0.1011 | No |
| 10 | AKT2 | na | 10210 | 0.092 | -0.1020 | No |
| 11 | STK11 | na | 11264 | 0.074 | -0.1259 | No |
| 12 | PTPN11 | na | 11569 | 0.069 | -0.1285 | No |
| 13 | PPARA | na | 11810 | 0.066 | -0.1296 | No |
| 14 | ACSL1 | na | 11845 | 0.066 | -0.1248 | No |
| 15 | AGRP | na | 12063 | 0.063 | -0.1255 | No |
| 16 | RXRA | na | 12183 | 0.061 | -0.1236 | No |
| 17 | MAPK10 | na | 12440 | 0.057 | -0.1259 | No |
| 18 | ADIPOR1 | na | 12737 | 0.053 | -0.1297 | No |
| 19 | IKBKB | na | 12994 | 0.050 | -0.1327 | No |
| 20 | TRAF2 | na | 13353 | 0.046 | -0.1390 | No |
| 21 | PRKCQ | na | 13573 | 0.043 | -0.1415 | No |
| 22 | PRKAG1 | na | 13773 | 0.041 | -0.1437 | No |
| 23 | PRKAG3 | na | 14342 | 0.034 | -0.1570 | No |
| 24 | CHUK | na | 14348 | 0.034 | -0.1542 | No |
| 25 | TRADD | na | 15689 | 0.021 | -0.1910 | No |
| 26 | CPT1C | na | 16818 | 0.010 | -0.2226 | No |
| 27 | PRKAA1 | na | 17163 | 0.007 | -0.2319 | No |
| 28 | AKT1 | na | 18101 | -0.001 | -0.2588 | No |
| 29 | MAPK8 | na | 18612 | -0.006 | -0.2730 | No |
| 30 | ACACB | na | 18637 | -0.007 | -0.2731 | No |
| 31 | ACSL4 | na | 19912 | -0.021 | -0.3080 | No |
| 32 | ADIPOR2 | na | 20619 | -0.029 | -0.3258 | No |
| 33 | TNFRSF1B | na | 21274 | -0.037 | -0.3415 | No |
| 34 | PRKAB2 | na | 21337 | -0.037 | -0.3399 | No |
| 35 | IKBKG | na | 21687 | -0.042 | -0.3463 | No |
| 36 | CAMKK2 | na | 22187 | -0.048 | -0.3564 | No |
| 37 | JAK2 | na | 23139 | -0.062 | -0.3784 | No |
| 38 | TNFRSF1A | na | 23281 | -0.064 | -0.3768 | No |
| 39 | CD36 | na | 23373 | -0.065 | -0.3737 | No |
| 40 | G6PC2 | na | 24433 | -0.083 | -0.3969 | No |
| 41 | ACSL5 | na | 25278 | -0.098 | -0.4126 | Yes |
| 42 | ACSL3 | na | 25434 | -0.102 | -0.4081 | Yes |
| 43 | NFKBIB | na | 25462 | -0.102 | -0.3999 | Yes |
| 44 | RXRB | na | 25902 | -0.111 | -0.4028 | Yes |
| 45 | PRKAB1 | na | 26497 | -0.123 | -0.4090 | Yes |
| 46 | STAT3 | na | 26744 | -0.129 | -0.4047 | Yes |
| 47 | LEPR | na | 27355 | -0.144 | -0.4096 | Yes |
| 48 | PPARGC1A | na | 27443 | -0.146 | -0.3992 | Yes |
| 49 | CPT1B | na | 27722 | -0.153 | -0.3937 | Yes |
| 50 | SLC2A1 | na | 27886 | -0.157 | -0.3845 | Yes |
| 51 | NFKB1 | na | 28241 | -0.168 | -0.3799 | Yes |
| 52 | PCK1 | na | 28560 | -0.177 | -0.3735 | Yes |
| 53 | SLC2A4 | na | 29009 | -0.191 | -0.3695 | Yes |
| 54 | SOCS3 | na | 29100 | -0.194 | -0.3549 | Yes |
| 55 | PRKAA2 | na | 29444 | -0.204 | -0.3468 | Yes |
| 56 | POMC | na | 30351 | -0.236 | -0.3521 | Yes |
| 57 | PCK2 | na | 30749 | -0.252 | -0.3413 | Yes |
| 58 | RELA | na | 31135 | -0.271 | -0.3285 | Yes |
| 59 | NFKBIA | na | 31525 | -0.292 | -0.3139 | Yes |
| 60 | IRS2 | na | 32897 | -0.380 | -0.3199 | Yes |
| 61 | NFKBIE | na | 32930 | -0.382 | -0.2871 | Yes |
| 62 | NPY | na | 33934 | -0.510 | -0.2710 | Yes |
| 63 | TNF | na | 34328 | -0.609 | -0.2285 | Yes |
| 64 | RXRG | na | 34332 | -0.610 | -0.1748 | Yes |
| 65 | AKT3 | na | 34696 | -0.932 | -0.1030 | Yes |
| 66 | IRS1 | na | 34720 | -1.176 | 0.0002 | Yes |
Table: GSEA details [plain text format]

  

Fig 2: KEGG\_ADIPOCYTOKINE\_SIGNALING\_PATHWAY      
 Blue-Pink O' Gram in the Space of the Analyzed GeneSet

  

Fig 3: KEGG\_ADIPOCYTOKINE\_SIGNALING\_PATHWAY: Random ES distribution      
 Gene set null distribution of ES for **KEGG\_ADIPOCYTOKINE\_SIGNALING\_PATHWAY**
